# Impact of Chirality on the Dynamic Susceptibility of Concentric Nanotori

**DOI:** 10.3390/nano15130989

**Published:** 2025-06-26

**Authors:** Ulises Guevara, Eduardo Saavedra, Liliana Pedraja-Rejas, Miguel-Angel Garrido-Tamayo, Solange Aranzubia, Eduardo Cisternas, Pablo Díaz, David Laroze

**Affiliations:** 1 Vicerrectoría de Investigación y Postgrado, Universidad de La Serena, La Serena 1700000, Chile; ujguev@gmail.com; 2 Departamento de Física, Universidad de Santiago de Chile (USACH), Santiago 9170124, Chile; 3 Departamento de Ingenería Industrial y de Sistemas, Universidad de Tarapacá, Casilla 7D, Arica 1000000, Chile; lpedraja@academicos.uta.cl; 4 Escuela de Física, Universidad Nacional de Colombia, Medellín 050034, Colombia; magarridot@unal.edu.co; 5 Departamento de Matemáticas, Universidad de Tarapacá, Casilla 7D, Arica 1000000, Chile; sranzubia@academicos.uta.cl; 6 Departamento de Ciencias Físicas, Universidad de La Frontera, Temuco 4811230, Chile; eduardo.cisternas@ufrontera.cl (E.C.); pablo.diaz@ufrontera.cl (P.D.); 7 Instituto de Alta Investigación, Universidad de Tarapacá, Casilla 7D, Arica 1000000, Chile; dlarozen@academicos.uta.cl

**Keywords:** permalloy, toroids, dynamic susceptibility, micromagnetism, micromagnetic simulation, Mumax3

## Abstract

This study investigates the influence of chirality on the dynamic susceptibility of concentric nanotori via micromagnetic simulations. The aim is to analyze the ferromagnetic resonance characteristics of coupled nanotori structures and compare them across various ring separation distances, thus providing an insight into how vortex configurations with identical or differing chiralities affect their dynamic properties. We analyze the energetic differences between the two vortex configurations and find them to be negligible; however, these minor differences suffice to explain the significant discrepancies in the demagnetization field observed between the nanotori. We examine the dynamic susceptibility spectrum and the spatial localization of the ferromagnetic resonance modes for different nanotori separations. Our findings demonstrate that the resonant oscillation frequencies are significantly influenced by the magnetostatic interactions between the nanotori, which can be effectively modulated by varying the distance between them. Furthermore, for smaller separations, the frequency peaks in the dynamic susceptibility markedly diverge between the two vortex configurations, demonstrating that the observed differences in the demagnetization field between the rings strongly influence the frequency response. In summary, our results indicate that both the inter-ring distance and the vortex configuration play a crucial role in determining the frequency response of the system.

## 1. Introduction

Low-dimensional magnetic structures have attracted significant attention as promising candidates for quantum magnetic memory applications [1,2,3,4,5,6,7,8,9,10]. These systems function as magnetic memories by enabling controlled topological transitions, primarily between two well-defined magnetization states, which are stabilized by geometric confinement. For example, the polarity of magnetic vortices in nanoscale disks, whose dynamics are predominantly confined to the material plane, can be dynamically manipulated through spin-wave (SW) excitation [11]. Recent studies in this area include [12], which analyzed vortex reversal induced by surface acoustic waves, and [13,14], which examined the influence of perpendicular uniaxial anisotropy on the switching of a magnetic vortex. A comprehensive review summarizing the current state of magnetic vortices and their potential applications can be found in [15,16,17]. Additional analyses of magnetic susceptibility in nanostructures suggest that both the resonance frequencies and the number of peaks are dependent on the geometric shape, which exerts a significant impact on the resonance behavior [1,3,18].

The emergence of three-dimensional (3D) magnetic structures, such as nanocylinders, nanowires, and nanotubes, introduces an additional spatial dimension that significantly affects magnetic properties. These structures are promising for spintronic applications, including racetrack memory and logic devices [19,20], due to their topological protection, which enhances stability and preserves magnetization profiles. Magnetic nanotori are of particular interest as they more effectively minimize stray field effects compared to cylindrical nanorings [21,22]. Vojkovic et al. (2016) [23] explored the magnetization states of toroidal nanomagnets, identifying vortex and in-plane single-domain configurations as ground states. In 2017, they demonstrated that curvature-induced chiral interactions lead to vortex and antivortex remanent states [24]. Mishra et al. (2017) [25] studied magnetization reversal in soft permalloy toroidal rings, finding that thin rings adopt an onion state, while thicker rings exhibit a stable vortex state. Lewis et al. (2020) [26] produced a phase diagram predicting stable vortex remanent states in magnetite nanotori, even for nanoparticles as small as 60 nm. XianYu et al. (2020) [27] analyzed the dynamic susceptibility spectra of toroidal nanorings under a canted DC magnetic field, observing natural vortex formation. Recently, Corona et al. (2023) [28] examined the stability and dynamics of a hopfion configuration in a toroidal nanoring under a uniaxial magnetic field. Saavedra et al. (2024) [29] investigated the static and dynamic responses of hopfions in cylindrical and toroidal nanorings under external magnetic fields. These findings underscore the interplay between curvature, topology, and magnetization, establishing nanotori as key platforms for studying complex magnetic phenomena.

Given the significance of spin waves, vortex configurations, and nanotorus-shaped nanostructures for various applications, this study investigates the dynamic behavior of concentric nanotori featuring vortices with identical and opposite chirality. Through micromagnetic simulations, we explore the susceptibility spectrum as a function of the system’s geometric parameters. The ferromagnetic resonance of coupled nanotori is analyzed and compared across different ring separation distances to elucidate the impact of these configurations on dynamic properties. Our results reveal promising trends that could inform the design of next-generation spintronic devices, enhancing data storage and processing efficiency.

## 2. Micromagnetic Simulations

Micromagnetic simulations were performed using the open-source software MuMax3 [30], which solves the Landau–Lifshitz–Gilbert equation using the finite difference method in time:

(1) $$\frac{d\vec{m}}{dt}=-\gamma \;\left(\vec{m}\times {\vec{H}}_{\mathrm{eff}}\right)+\alpha \left(\vec{m}\times \frac{d\vec{m}}{dt}\right)\;$$

where α and γ represent the Gilbert damping constant and the gyromagnetic ratio, respectively. $\vec{m}=\vec{M}/M_{s}$ denotes the normalized magnetization vector, $M_{s}$ is saturation magnetization, and ${\vec{H}}_{\mathrm{eff}}$ is the effective field

(2) $${\vec{H}}_{\mathrm{eff}}=-\left(1/\mu_{0}M_{s}\right)\delta \varepsilon /\delta \vec{m}$$

$\varepsilon =\varepsilon_{ex}+\varepsilon_{d}+\varepsilon_{Z}$ is the total energy density, with $\left(\varepsilon_{ex},\varepsilon_{d},\varepsilon_{Z}\right)$ being the exchange, dipolar, and Zeeman energies, respectively. Each energy density is expressed as follows:

(3) $$\begin{array}{c} \varepsilon_{ex}=A\left[{\left(\vec{\nabla }m_{x}\right)}^{2}+{\left(\vec{\nabla }m_{y}\right)}^{2}+{\left(\vec{\nabla }m_{z}\right)}^{2}\right] \end{array}$$

(4) $$\begin{array}{c} \varepsilon_{d}=\frac{1}{2}\mu_{0}M_{s}\left(\vec{m}\cdot \vec{\nabla }U\right) \end{array}$$

(5) $$\begin{array}{c} \varepsilon_{Z}=-\mu_{0}\left(\vec{m}\cdot \vec{H}\right) \end{array}$$

where *A* and $\mu_{0}$ are the exchange constant and the magnetic permeability of the vacuum, $\vec{m}$ is magnetization, and $\vec{H}$ is the applied external magnetic field. The term *U* is the scalar magnetic potential, which is defined as

(6) $$\begin{array}{c} U=\frac{1}{4\pi }\oint_{S}\frac{{\vec{n}}^{\prime }\cdot {\vec{m}}^{\prime }}{|{\vec{r}}^{\prime }-\vec{r}|}\;dS^{\prime }-\frac{1}{4\pi }\int_{V}\frac{{\vec{\nabla }}^{\prime }\cdot {\vec{m}}^{\prime }}{|{\vec{r}}^{\prime }-\vec{r}|}\;dV^{\prime } \end{array}$$

in which the first term represents the contribution of magnetic moments at the surface, and the second term represents the contribution of magnetic moments throughout the volume of the material. Here, ${\vec{n}}^{\prime }$ denotes the unit normal vector to the surface, ${\vec{r}}^{\prime }$ and $\vec{r}$ arethe position vectors of the source and field points, respectively, $S^{\prime }$ is the surface enclosing the region of interest, and *V* is the volume of the magnetic body.

**Figure 1:**
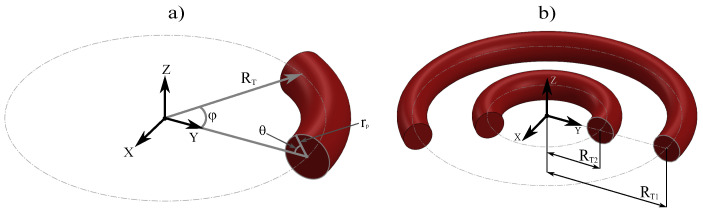
(**a**) Geometric parameters defining a toroid, where $R_{T}$ is the toroidal radius, $r_{p}$ the poloidal radius, θ the poloidal angle, and φ the azimuthal angle in the xy-plane. (**b**) System of coplanar nanotori. $R_{T2}$ represents the toroidal radius of the inner toroid, and $R_{T1}$ represents the toroidal radius of the outer toroid. In this system, the parameters $r_{p}$, θ, and φ are the same for both nanotori.

The construction of any toroid on the xy plane can be carried out using the following equation:

(7) $$\begin{array}{c} \vec{r_{T}}=\left[R_{T}-r_{p}\cos\left(\theta \right)\right]\cos\left(\varphi \right)\hat{x}\\ +\left[R_{T}-r_{p}\cos\left(\theta \right)\right]\sin\left(\varphi \right)\hat{y}\\ +r_{p}\sin\left(\theta \right)\hat{z} \end{array}$$

where $\vec{r_{T}}$ is the position vector of any point on the toroid, $R_{T}$ is the toroid radius, measured from the origin of the coordinate system to the central axis of the toroid, $r_{p}$ is the poloidal radius, measured from the central axis of the toroid and indicating its thickness, θ is the poloidal angle, located in the plane of the poloidal radius, and φ is the azimuthal angle, located in the xy-plane of the coordinate system, as shown in Figure 1a.

With these parameters, we will construct a system of two concentric nanotori, which are described as follows: a fixed toroidal radius $R_{T1}=226$ nm for the outermost toroid, and a variable toroidal radius $R_{T2}$ for the second toroid.

$$\begin{array}{c} R_{T2}=\delta \left(R_{T1}-3r_{p}\right)+r_{p} \end{array}$$

**Figure 2:**
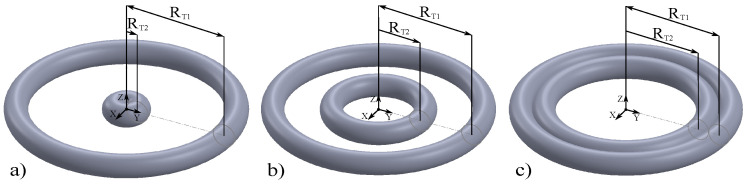
(**a**) The parameter δ indicates the variation in the toroidal radius of the inner toroid. For δ=0.0, $R_{T2}=24$ nm; for (**b**) δ=0.5, $R_{T2}=89$ nm; for (**c**) δ=1.0, $R_{T2}=154$ nm.

The poloidal radius $r_{p}=24$ nm, the poloidal angle $\theta_{p}$ and the azimuthal angle $\varphi_{T}$ located in the xy plane are the same for both nanotori, as shown in Figure 1b. Here, δ is a proportionality factor defined between 0.0 for $R_{T2}=r_{p}$ and 1.0 for $R_{T2}=R_{T1}-2r_{p}$, so that the inner toroid only touches the surface of the outer toroid, as seen in Figure 2c.

Permalloy is a soft ferromagnetic material known for its exceptional magnetic properties, including high permeability, low coercivity, and near-zero magnetostriction [31]. These characteristics make it an ideal candidate for fundamental studies where the aim is to isolate and analyze the influence of geometry on magnetic behavior. In this context, numerical simulations were performed using standard Py material parameters [8,29]: a saturation magnetization of $M_{s}=800\times 10^{3}$ A$m^{-1}$, and an exchange stiffness constant of $A=1.30\times 10^{-11}$ J $m^{-1}$. Polycrystalline samples were considered, so the magnetocrystalline anisotropy K was not taken into account. The system is discretized using a fine mesh size of 2×2×2 n$m^{3}$ to accurately represent a smooth geometry while minimizing border effects [29,32]. Additional calculations were performed with 1×1×1 n$m^{3}$ cells, but no appreciable changes were observed, with the difference being less than 1% (see Figure A4 in the Appendix A).

We employed the ringdown method to investigate the resonant modes of vortices in magnetic concentric nanotori. The initial magnetic configuration was established, and the final state was obtained by integrating the Landau–Lifshitz–Gilbert (LLG) equation until the torque dropped below $10^{-4}$ T [33,34]. After convergence, a 100 ns minimization step was applied to dampen spin waves, stabilizing the configuration [35]. Throughout this entire process, we utilized a damping parameter of α = 0.5, which is a common practice in micromagnetic simulations and does not introduce significant deviations in the results [36]. To analyze the dynamical susceptibility, we adopted a lower damping value of α = 0.008 [37,38,39].

To obtain the dynamical response of the magnetization, we simulated the system’s magnetization dynamics under the action of a sinc field, expressed as $h\left(t\right)=h_{0}\;\mathrm{sinc}\left\{2\pi f_{\max}\left(t-t_{0}\right)\right\}\;x$ [36,40], with $h_{0}=1$ mT, f=25 GHz, and $t_{0}=1$ ns. The pulse amplitude was sufficiently small to maintain the system within the linear response regime [41]. Magnetization dynamics were tracked for 100 ns, with configurations recorded at uniform intervals of 20 ps, providing a spectral resolution of 0.01 GHz. The Fourier transform of the sinc function in the time domain resulted in a rectangular function in the frequency domain. This implies that modes above the cutoff frequency ($f_{\max}$) are significantly attenuated (and ideally non-existent) in the response spectrum [42]. The excitation field h(t) and magnetization M(r,t) were transformed into the frequency domain, [h(ω),M(ω)], using a Fast Fourier Transform (FFT). The dynamic susceptibility, corresponding to the imaginary part of the magnetic susceptibility, was calculated as the ratio M(ω)/h(ω) [43,44]. Finally, to confirm the origin of the resonant peaks, we reconstructed the spatial profiles of the resonant modes by calculating the temporal Fourier image for each site as $\tilde{m}\left(r_{ijk},f_{n}\right)={\mathrm{DFT}}_{t}\left\{m\left(r_{ijk},t\right)\right\},$ where DF$T_{t}$ is the Discrete-time Fourier Transform, the subscript ijk corresponds to the spatial coordinates x,y,z of each cell, and the subscript *n* indicates the frequency index in the power spectrum.

## 3. Results

**Figure 3:**
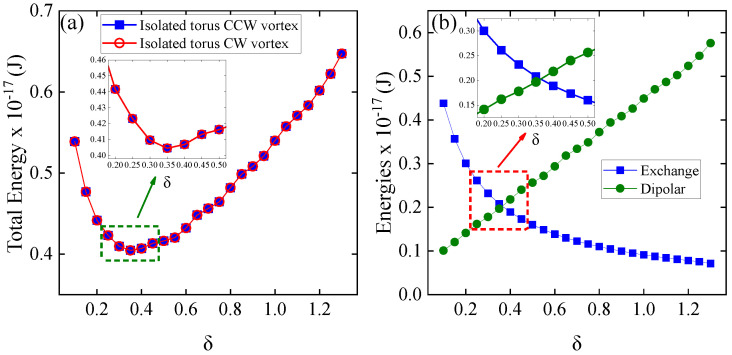
(**a**) Total energy of an isolated nanotorus as a function of the toroidal radius parameter, δ, for both counterclockwise (CCW) and clockwise (CW) vortex configurations. The CCW vortex is represented by blue squares, while the CW vortex is represented by red circles. The inset provides a zoomed-in view of the energy minimum near δ=0.35. (**b**) The graph separates the energy contributions, divided into exchange and dipolar contributions, with exchange energy shown by blue squares and dipolar energy by green circles.

The first system studied is an isolated nanotorus with a fixed poloidal radius of 50 nm, where the toroidal radius is systematically varied using a δ parameter from 0.1 to 1.3 in increments of 0.05. This setup provides a powerful platform for analyzing vortex-related energetic processes and dynamic magnetic properties. We began our simulations with an initial vortex configuration. Figure 3 presents the energy profile of an isolated nanotorus with a fixed poloidal radius of 50 nm as a function of the toroidal radius parameter, δ, which ranges from 0.1 to approximately 1.3. Figure 3a illustrates the total energy for both counterclockwise (CCW) and clockwise (CW) vortex configurations. Notably, both configurations demonstrate identical behavior, with the total energy decreasing to a minimum at δ = 0.35 before subsequently increasing. The inset provides a detailed look at this critical region. Figure 3b divides the total energy into its exchange and dipolar contributions. As δ increases, the exchange energy (represented by blue squares) sharply decreases, whereas the dipolar energy (indicated by green circles) almost linearly increases. The inset clearly highlights the balance between these competing interactions at δ=0.35. Vojkovic et al. (2016) [23] observed a comparable trend, linking the continuous dipolar energy to small magnetic charges resulting from the toroid discretization process. In their work, a slight decrease in exchange energy was observed, likely due to small deviations in the magnetic moments. In our study, varying the R/r ratio from 1.64 to 9.34 led to a crossover between dipolar and exchange energy contributions, pinpointing the minimum total energy. Indeed, we observed a pronounced decrease in exchange energy with increasing δ, since a larger nanotorus radius makes the moments more collinear, thus reducing the energy as the system approaches a ferromagnetic state.

**Figure 4:**
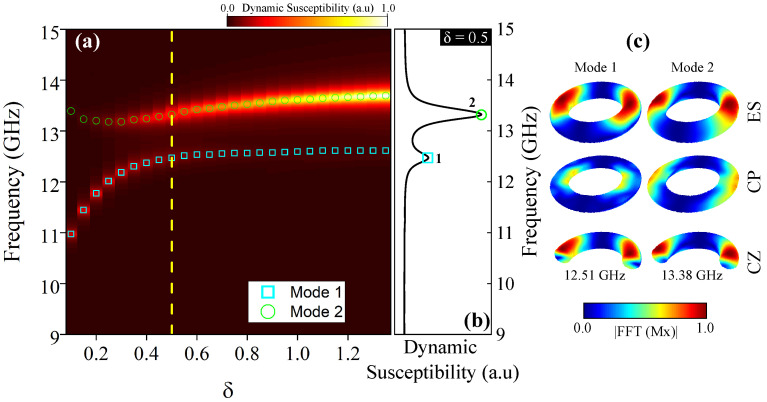
(**a**) Frequency of the resonant modes as a function of δ parameter for an isolated nanotorus with a fixed poloidal radius of 50 nm. Two distinct resonance modes are observed: mode 1 (squares) and mode 2 (circles). (**b**) Dynamic susceptibility profile (yellow dashed line in Figure 4a) at δ=0.5, showing the relative amplitude of modes 1 and 2. (**c**) Spatial distribution of the resonant modes for δ=0.5. The color code represents the amplitude of the FFT applied to the x-component of the magnetization in the external surface (ES), central plane (CP) and the z-axis direction (CZ), where red indicates a higher spin amplitude and blue signifies zero spin amplitude.

In Figure 4, we present and analysis of the frequency modes of a magnetic vortex in a nanotorus using a sinc field. The frequency range spans 0–15 GHz at H=0 mT, revealing two distinct modes, mode 1 and mode 2, which are both dependent on δ. All frequencies fell between 9 GHz and 14 GHz. As seen in Figure 4a, mode 1 increases from 10.97 GHz to 12.61 GHz as δ varies from 0.1 to 1.3, while mode 2 shifts slightly, from 13.39 GHz to 13.69 GHz. Mode 1 corresponds to a low-energy, low-amplitude response, whereas mode 2 exhibits a higher-energy state with greater amplitude, as shown in Figure 4b for δ=0.5. To clarify the origin of these resonant modes, Figure 4c illustrates their spatial distribution for δ=0.5. The columns represent resonance modes, while the rows display the nanotorus external surface (ES) and the central plane (CP), which correspond to cross-sections along the central xy-plane and the cross-sections in the z-axis direction (CZ). Resonance modes 1 and 2 mainly arose from the excitation of magnetic moments on the ES, exhibiting 1 and 2 nodes along the CS, respectively. Additionally, mode 1 features localized internal surface excitations, while mode 2 extends further across the structure. Our results align with prior findings [27,28], where two peaks in the dynamic susceptibility of the nanotorus are observed.

**Figure 5:**
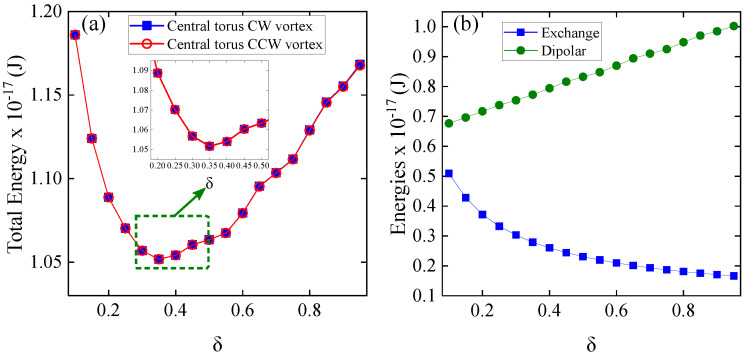
(**a**) Total energy of the concentric nanotori system is shown as a function of δ, with the outer torus fixed in a counterclockwise (CCW) vortex configuration and the inner torus vortex chirality varying between counterclockwise (CCW) and clockwise (CW). The inset provides a zoomed-in view of the energy minimum near δ=0.35. (**b**) The graph separates the energy contributions into exchange energy (blue squares) and dipolar energy (green circles).

**Figure A1:**
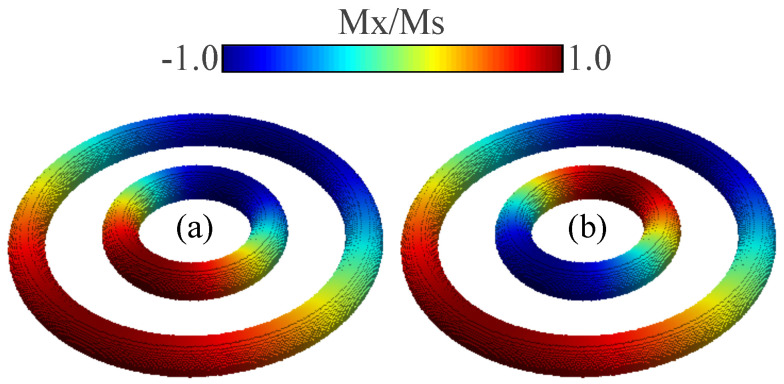
Magnetization profiles of concentric nanotorus rings, where the outer torus consistently hosts a counterclockwise (CCW) vortex. (**a**) Configuration in which the inner torus shares the same chirality, exhibiting a counterclockwise (CCW) vortex. (**b**) Configuration in which the inner torus has opposite chirality, displaying a clockwise (CW) vortex. The color scale represents the normalized x-component of the magnetization (Mx/Ms), with red indicating positive maximum magnetization and blue indicating negative maximum magnetization.

Now, we examine a system of two concentric nanotori, consisting of an outer nanotorus with a fixed toroidal radius (δ=1.3, equivalent to $R_{T1}=226$ nm) and an inner nanotorus with a variable toroidal radius. The δ parameter of the inner nanotorus varied from 0.1 (non-interacting nanotori, $R_{T2}=40$ nm) to 0.95 (interacting nanotori, $R_{T2}=170$ nm) in increments of 0.05. The poloidal radius for both nanotori was consistently set at 50 nm. In our simulations, we analyzed two configurations based on the initial chirality of the vortices: the same or opposite chirality. For all cases, the outer torus hosts a counterclockwise (CCW) vortex. In contrast, the chirality of the inner torus varied between counterclockwise (same chirality) and clockwise (opposite chirality); see Figure A1 in the Appendix A. In Figure 5, we illustrate the energy behavior of the concentric nanotori system for both cases. In the left graph, the total energy is shown. Both cases exhibit a similar trend, where the energy decreases to a minimum of around δ=0.35 before increasing. The inset zooms in on the energy values around this minimum, highlighting the detailed energy landscape near δ=0.35. A closer look (see Figure A3 in the Appendix A) shows that the total energy is higher in the CW-CCW case than in the CCW-CCW case, with the difference accentuated when the nanotori are closer (higher δ values). Although the energy difference is slight, it shows that the interaction slightly affects the magnetic texture of the vortices. In the right graph, the energy contributions are separated into exchange energy (blue squares) and dipolar energy (green circles), illustrating how these components vary as the value of δ increases (only the case of equal chirality is shown, as the other case is very similar). The dipolar energy consistently remains higher than the exchange energy as δ increases, due to the long-range nature of dipolar interactions [18,23,45], which are amplified by the external toroidal geometry. The exchange energy remains lower, as this primarily involves local interactions. Comparing Figure 5b with Figure 3b, both have the same characteristics. Moreover, the variations in the dipole and exchange energies between δ=0.1 and 1.0 are practically the same. This is not surprising, as Figure 5a shows that the energy difference between the magnetic configurations is very small, revealing the weak interaction between the nanotori. This weak interaction means that the magnetic configuration of the outer nanotorus remains almost invariant throughout the δ range, and only the inner nanotorus energy varies due to the change in radius (and volume), with a similar behavior to that of a single nanotorus. Although the energy difference is small, it will significantly influence the dynamic susceptibility.

**Figure 6:**
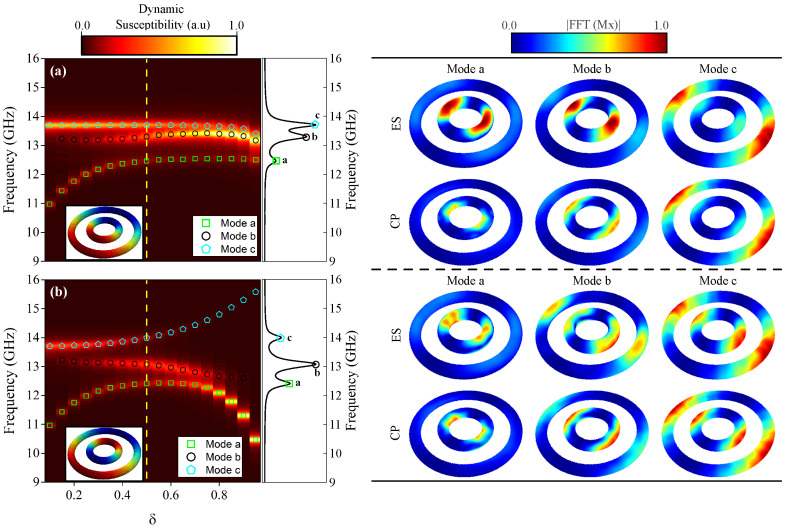
(**a**) The frequency of the resonant modes as a function of the δ parameter for concentric nanotori, where the central torus has a counterclockwise (CCW) vortex, matching the chirality of the outer torus. (**b**) The frequency of the resonant modes as a function of δ parameter for concentric nanotori where the central torus has a clockwise (CW) vortex, resulting in opposite chirality compared to the outer torus. The modes are labeled as “a”, “b”, and “c”, with dynamic susceptibility shown in the vertical middle panel, corresponding to the yellow dashed lines in (**a**,**b**). The right panels illustrate the spatial distributions of the resonant modes for δ=0.5. The color code represents the amplitude of the FFT applied to the x-component of the magnetization in the external surface (ES) and central plane (CP), where red indicates a higher spin amplitude and blue signifies a zero spin amplitude.

**Figure A2:**
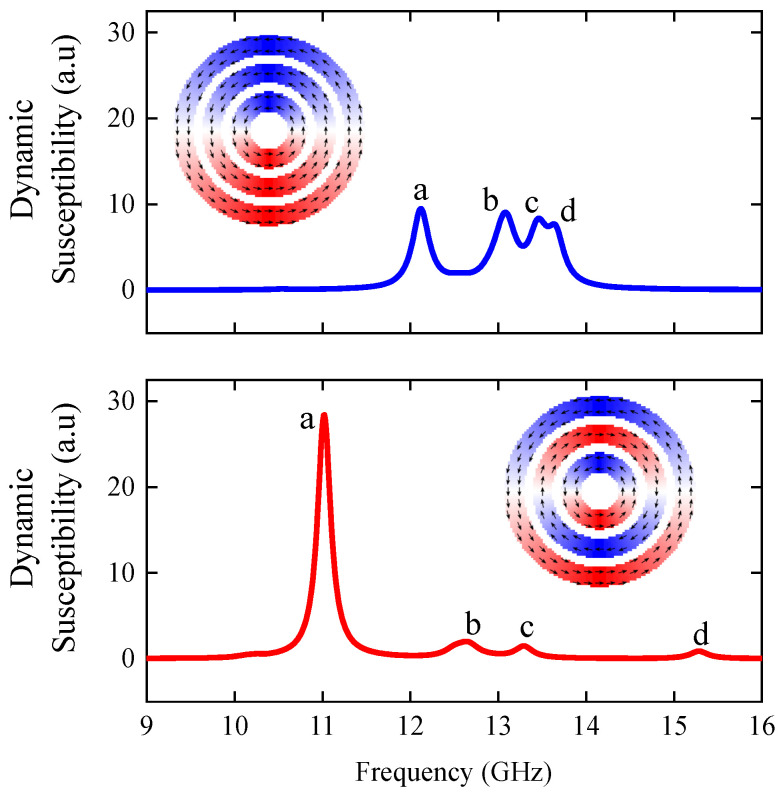
(**Top**) Dynamic susceptibility spectra for three concentric nanotori housing a vortex with identical chirality, displaying four resonant modes labeled as “a”, “b”, “c”, and “d”. (**Bottom**) Dynamic susceptibility spectra for three concentric nanotori housing a vortex with differing chiralities, revealing four resonant modes also labeled as “a”, “b”, “c”, and “d”.

**Figure A3:**
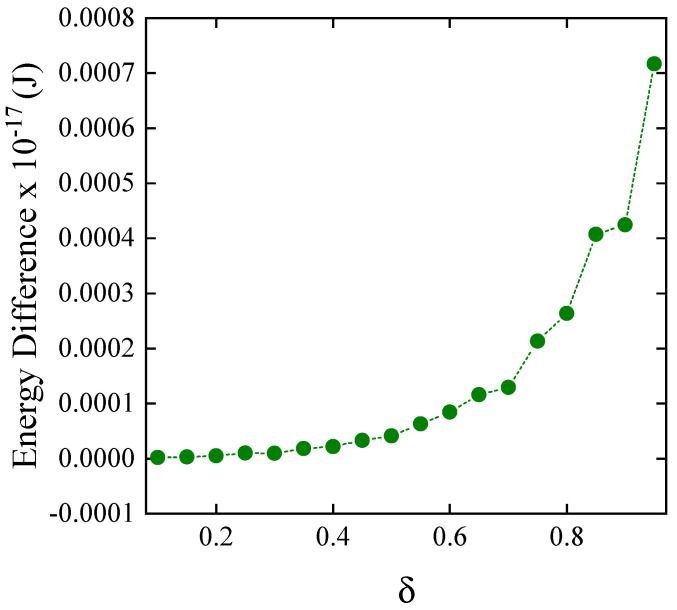
Energy difference between two concentric nanotori exhibiting the CW-CCW and CCW-CCW cases.

**Figure A4:**
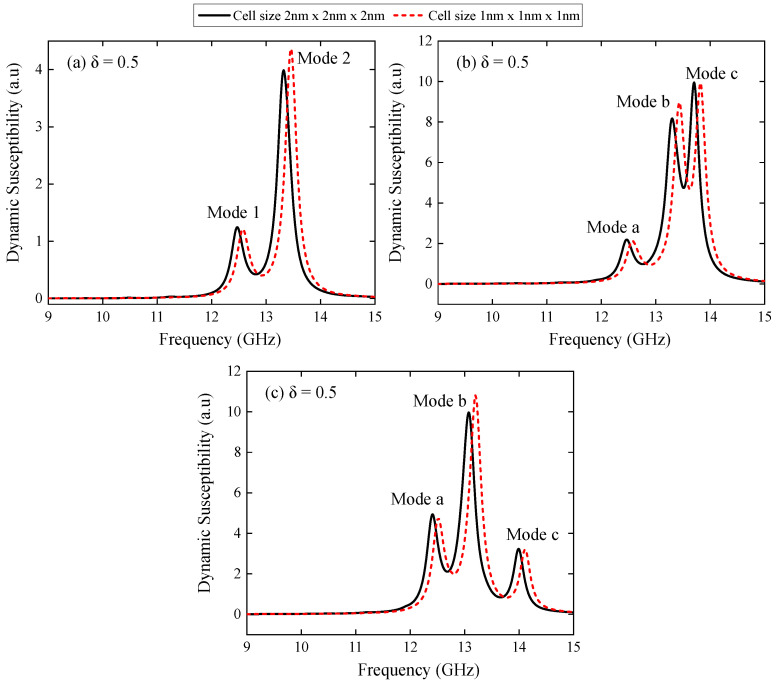
Comparative dynamic susceptibility spectra for δ=0.5 corresponding to: (**a**) isolated nanotorus, (**b**) concentric nanotori with identical chirality, and (**c**) concentric nanotori with opposite chirality. Distinct resonance modes are labeled, and results are shown for simulations with cell sizes of 2 nm × 2 nm × 2 nm and 1 nm × 1 nm × 1 nm.

Figure 6a,b illustrate the frequency response of two concentric nanotori as a function of the parameter δ, covering a frequency range from 9 to 16 GHz at H=0 mT. We identify three distinct modes, labeled “a”, “b”, and “c”, which depend directly on δ. It is important to note that if we had three concentric toroidal rings, they would exhibit four resonant modes (see Figure A2 in the Appendix A). Specifically, Figure 6a presents results for both nanotori with counterclockwise (CCW) vortices (the same chirality). In contrast, Figure 6b shows the outer nanotorus with a CCW vortex and the inner nanotorus with a clockwise (CW) vortex (opposite chirality). In both configurations, mode “a” appears around 10.96 GHz, mode “b” around 13.24 GHz, and mode “c” around 13.70 GHz for δ = 0.0, although each mode exhibits a different behavior as δ varies. The resonance modes converge to a frequency region between 12 GHz and 14 GHz for concentric nanotori with the same chirality. However, in the case of opposite chirality, the modes diverge, with resonance frequencies spanning from 9 GHz to 16 GHz. To gain a deeper understanding of this, we analyzed the spatial distribution of resonance modes “a”, “b”, and “c” at a representative δ=0.5 for each chirality configuration, as shown in the right panels, with views labeled ES (External Surface) and CP (Central Plane). Mode “a” exhibits localized magnetization oscillations on the ES, with some of the oscillations in the magnetic moments concentrated in the inner part of the inner nanotorus (see CP view), resembling resonance mode “1” in an isolated nanotorus. Mode “b” shows localized oscillations on the ES, with some of the oscillations concentrated in the outer part of the inner nanotorus (see CP view), similar to resonance mode “2” in an isolated torus. Mode “c” primarily displays oscillations on the ES, with oscillations in the magnetic moments concentrated in the outer part of the outer nanotorus (see CP view).

**Figure 7:**
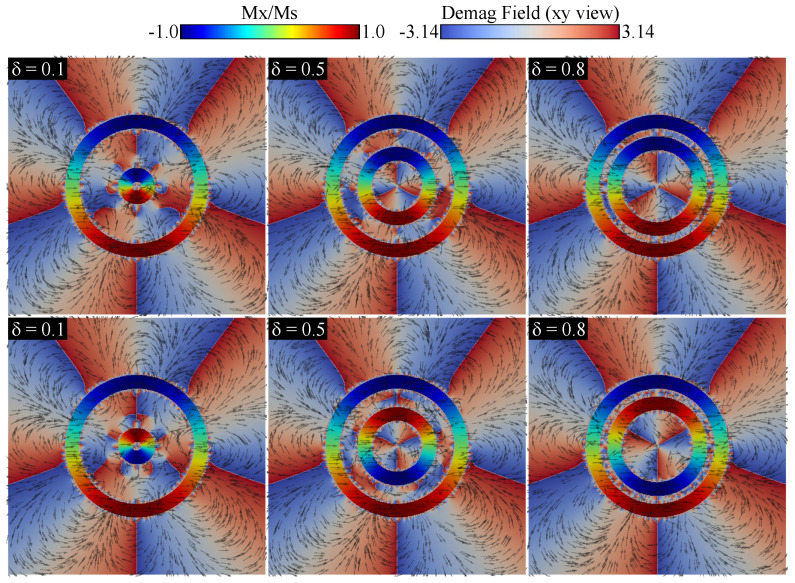
(**Top**) Demagnetizing field for a configuration where the inner nanotorus shares the same chirality, exhibiting a counterclockwise (CCW) vortex with parameter δ=0.1, 0.5, and 0.8. (**Bottom**) Demagnetizing field for a configuration where the inner nanotorus has opposite chirality, displaying a clockwise (CW) vortex with parameter δ=0.1, 0.5, and 0.8.

This difference in dynamic magnetic response at the frequencies of resonant modes when two configurations are present has been reported in another nanostructure. Yang et al. [46] investigated spin-dynamic modes in cylindrical nanotubes with vortex chirality configurations at the ends, showing that these configurations significantly affect the dispersion of standing spin waves (SWs). The nonreciprocal propagation of SWs was attributed to dynamic dipolar fields in an azimuthally magnetized ferromagnetic tube, as reported by Talora et al. [47]. McKeever et al. [6] observed that when concentric permalloy rings with opposite chirality vortices are perturbed by pulse excitation, the demagnetizing field aligns the magnetization in an antiparallel direction in adjacent rings, leading to frequency beating and modulation. For our system, we determined the demagnetizing fields for three cases—δ=0.1, 0.5, and 0.8—as shown in Figure 7.

We observed that the demagnetization field inside the nanotori remained stable as the inner nanotorus grew in diameter, confirming that the magnetic texture of each nanotorus does not change significantly, as seen in Figure 5. However, the demagnetization field between the nanotori behaves differently for nanotori with the same chirality (top) versus those with opposite chirality (bottom). This difference in the demagnetization fields explains the variations in resonance peaks, which are accentuated when the inner nanotorus approaches the outer one. Despite there being no significant energetic changes, the difference in the demagnetization field leads to notable changes in the resonant modes, allowing for small changes in the inner nanotorus radius to cause significant frequency response changes based on the magnetic configuration.

## 4. Conclusions

In conclusion, this study investigated the dynamic behavior of concentric magnetic nanotori exhibiting vortex-type magnetic states, focusing on their dependence on chirality and variations in the inner nanotorus radius. Micromagnetic simulations revealed that the total energy of the concentric nanotori system is comparable for both chirality configurations, though slightly elevated for vortices with opposite chirality. Notably, the system’s energy is approximately double that of an isolated nanotorus, highlighting the significance of inter-ring interactions.

For concentric nanotori with the same chirality, resonant frequencies cluster around 13 GHz, while for opposite chirality, these frequencies split, ranging from 9 GHz to 16 GHz. Each mode exhibits distinct spatial characteristics that are influenced by geometry and magnetic interactions: low-frequency modes are localized in the inner nanotorus, whereas high-frequency modes are dominated by oscillations in the outer nanotorus.

These findings demonstrate that minor adjustments in the inner nanotorus radius, represented by parameter δ, can lead to substantial shifts in frequency response due to variations in the demagnetization field. Although the energy differences between chirality configurations are minimal, they result in significant variations in the dynamic susceptibility spectrum, presenting a promising avenue for the design of tunable nanomagnetic devices where resonant frequencies can be modulated by manipulating chirality and geometric parameters.

## Data Availability

Data will be made available on request.
